# Mental Distress, Label Avoidance, and Use of a Mental Health Chatbot: Results From a US Survey

**DOI:** 10.2196/45959

**Published:** 2024-04-12

**Authors:** Kristin Kosyluk, Tanner Baeder, Karah Yeona Greene, Jennifer T Tran, Cassidy Bolton, Nele Loecher, Daniel DiEva, Jerome T Galea

**Affiliations:** 1 Department of Mental Health Law & Policy University of South Florida Tampa, FL United States; 2 School of Social Work University of South Florida Tampa, FL United States

**Keywords:** chatbots, conversational agents, mental health, resources, screening, resource referral, stigma, label avoidance, survey, training, behavioral, COVID-19, pilot test, design, users, psychological distress, symptoms

## Abstract

**Background:**

For almost two decades, researchers and clinicians have argued that certain aspects of mental health treatment can be removed from clinicians’ responsibilities and allocated to technology, preserving valuable clinician time and alleviating the burden on the behavioral health care system. The service delivery tasks that could arguably be allocated to technology without negatively impacting patient outcomes include screening, triage, and referral.

**Objective:**

We pilot-tested a chatbot for mental health screening and referral to understand the relationship between potential users’ demographics and chatbot use; the completion rate of mental health screening when delivered by a chatbot; and the acceptability of a prototype chatbot designed for mental health screening and referral. This chatbot not only screened participants for psychological distress but also referred them to appropriate resources that matched their level of distress and preferences. The goal of this study was to determine whether a mental health screening and referral chatbot would be feasible and acceptable to users.

**Methods:**

We conducted an internet-based survey among a sample of US-based adults. Our survey collected demographic data along with a battery of measures assessing behavioral health and symptoms, stigma (label avoidance and perceived stigma), attitudes toward treatment-seeking, readiness for change, and technology readiness and acceptance. Participants were then offered to engage with our chatbot. Those who engaged with the chatbot completed a mental health screening, received a distress score based on this screening, were referred to resources appropriate for their current level of distress, and were asked to rate the acceptability of the chatbot.

**Results:**

We found that mental health screening using a chatbot was feasible, with 168 (75.7%) of our 222 participants completing mental health screening within the chatbot sessions. Various demographic characteristics were associated with a willingness to use the chatbot. The participants who used the chatbot found it to be acceptable. Logistic regression produced a significant model with perceived usefulness and symptoms as significant positive predictors of chatbot use for the overall sample, and label avoidance as the only significant predictor of chatbot use for those currently experiencing distress.

**Conclusions:**

Label avoidance, the desire to avoid mental health services to avoid the stigmatized label of mental illness, is a significant negative predictor of care seeking. Therefore, our finding regarding label avoidance and chatbot use has significant public health implications in terms of facilitating access to mental health resources. Those who are high on label avoidance are not likely to seek care in a community mental health clinic, yet they are likely willing to engage with a mental health chatbot, participate in mental health screening, and receive mental health resources within the chatbot session. Chatbot technology may prove to be a way to engage those in care who have previously avoided treatment due to stigma.

## Introduction

Over 10 years ago, the Annapolis Coalition for Behavioral Health Workforce Development declared a behavioral health workforce crisis in a report summarizing the key resource challenges in the field [1]. Some of the key challenges noted in this report included the observation that behavioral health care workers are too few and poorly distributed throughout the country, leaving many communities without a trained behavioral health workforce. National projections for 2013-2025 suggest that behavioral health provider supply has not kept pace with demand [2]. Simply training more professionals will not be enough to address these issues of supply and demand [3].

In total, 5 key characteristics have been proposed for service delivery models that might effectively meet the demands placed on the behavioral health care system: (1) the capacity to reach individuals not usually served, (2) scalability (the ability to implement an intervention on a large scale), (3) affordability, (4) expansion of the nonlicensed (ie, peer and paraprofessional workforce via task shifting), and (5) expansion of settings (bringing interventions to locales where those in need are likely to participate) [4]. To a great extent, these 5 key characteristics can be addressed using technology [5-7].

One specific form of technology that holds great promise for addressing behavioral health care workforce demands is chatbot technology. Chatbots are conversational interfaces (eg, Amazon Alexa and customer service chatbots used by banks or insurance companies) that use text or speech in a conversational, human-like manner to deliver information. A major strength of bots is their centralized programming (ie, its “brains”) on secure, cloud-based computing servers, which permits users to interact with the chatbot via multiple, existing platforms like SMS texts, WhatsApp, and Facebook messenger without the need to install special software or apps. Chatbots are heavily used in consumer settings because of their ability to quickly provide tailored information and increase purchasing probability. In the health sector, chatbot use is less prevalent but has been applied to mental health intervention delivery [8,9].

Researchers and clinicians have argued for almost two decades that certain aspects of treatment can be removed from clinicians’ responsibilities and allocated to technology. Research suggests, for example, that some aspects of treatment, such as exposure for panic disorders or cognitive restructuring for depression can be delivered via technology with comparable outcomes to delivery by a clinician [10,11]. Such reallocations of clinical tasks can preserve valuable clinician time and should alleviate the burden on the behavioral health care system by only requiring that services tasks where efficacy is dependent on human interaction are delivered by a human. The most recent comprehensive review of the literature summarizing existing research on blended models of service delivery—models combining some aspects of face-to-face intervention with technology-based intervention [12]—categorized blending according to both the ratio of services delivered via technology versus face-to-face and the order in which the technology or face-to-face components were delivered. One type of sequential model presents internet intervention before face-to-face intervention to engage patients during wait times and uses stepped care to reduce clinician burden. Further, 2 of the studies included in the review showed significant differences in positive outcomes (ie, symptom reduction) between those individuals engaged via technology during waiting times versus those not engaged [13,14]. However, 1 study failed to show any difference between wait-list patients engaged via the internet before face-to-face intervention and patients engaged via a self-help booklet [15]. Unfortunately, none of the studies available at the time of this review examined the cost-effectiveness of this sequential blended service delivery model, though 1 published protocol describes plans to examine the cost savings of a stepped care model [16].

Several technology-based tools exist that deliver interventions using digital therapeutics. Examples of such tools include reSET-O (Digital Therapeutics Alliance) [17], a digital therapeutic for treating opioid use disorder, and Woebot (Woebot Health) [8] and Wysa (Wysa Ltd) [9], cognitive behavioral therapy–based conversational agents for addressing a range of clinical concerns among various populations. To our knowledge, less attention has been given to developing, testing, and using conversational agents (chatbots) for other clinical tasks. In addition to aspects of treatment, other service delivery tasks that could arguably be allocated to technology to alleviate clinician burden without negatively impacting patient outcomes include screening, triage to services of appropriate intensity (as in a stepped care model), and referral. This has been attempted in the field of substance abuse via the Substance Abuse and Mental Health Services Administration’s Screening, Brief Intervention, and Referral (SBIRT) program, which applies the principles of stepped care to substance abuse and aims to funnel people in need into substance use treatment [18]. Wouldes and colleagues [18] argue that technology-based delivery of SBIRT may be equally effective to human-delivered SBIRT and that technology-based delivery would increase the accessibility of this intervention and reduce the impact of stigma as a barrier to care for certain populations (eg, pregnant women using substances).

Here, we describe the results of a national survey. This survey was exploratory, designed to understand the relationship between potential mental health chatbot users’ demographics and actual chatbot use and uptake of mental health resources provided by a chatbot; the rate of completion of mental health screening when delivered by a chatbot; and the acceptability of a prototype chatbot designed for mental health screening and referral. This chatbot not only screened participants for psychological distress, but also referred them to appropriate resources (breathing exercises, self-help, peer resources, and crisis lines) that matched their level of distress and preferences. The goal of this study was to determine whether a mental health screening and referral chatbot would be feasible and acceptable to users. If indeed this type of technology is feasible and acceptable, it may hold promise to be tested in further research to help ease the strain on the behavioral health care system by assuming the role of screening and referral and delivering low-intensity interventions (eg, breathing exercises and self-help interventions) to those who are experiencing less distress, reserving referral to higher-intensity resources (eg, crisis lines and care from licensed clinicians) for those in greater distress. Additionally, conducting screening via chatbot technology may provide an opportunity to engage those who are currently experiencing significant distress, but are not yet willing to seek the help of a licensed clinician due to environmental barriers such as stigma, with some resources (eg, self-help or stigma reduction interventions) when otherwise they may receive no support.

## Methods

### Study Overview

During May and June 2021, we conducted a national, hybrid, cross-sectional, internet-based survey among a sample of US-based, English-speaking persons aged 18 years or older registered with the Amazon Mechanical Turk (MTurk) system; a subset of all survey users continued beyond the initial survey to interact with a prototype mental health chatbot and completed a second survey. Though not specifically designed as a research panel, MTurk has been increasingly used to recruit for research studies across social science disciplines [19] and is both more diverse and more attentive than college student samples [20]. Further, MTurk is largely representative of a broader population, skewing toward younger women of minority status [21].

**Figure 1:**
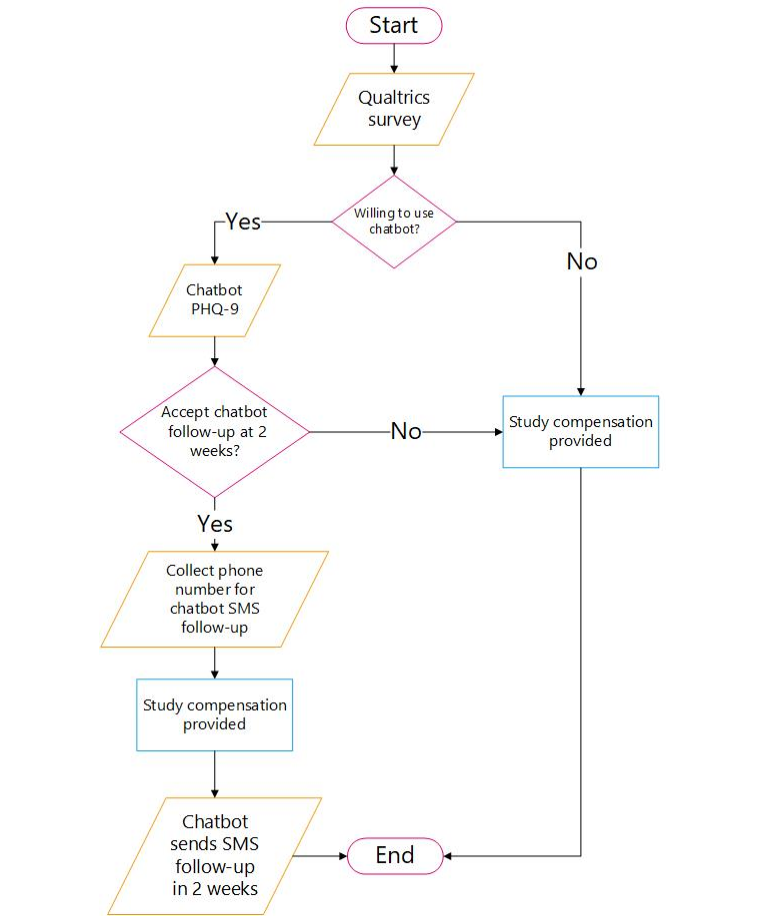
A diagram depicting procedures completed by all study participants. PHQ-9: Patient Health Questionnaire.

Figure 1 displays this study’s design. MTurk’s recruitment parameters were set to offer study participation only to users meeting the above inclusion criteria. Users interested in participating completed informed consent and then completed a web-based survey collecting demographic data and mental health history (from the PhenX toolkit) [22], along with a battery of measures assessing behavioral health and symptoms, stigma (label avoidance and perceived stigma), attitudes toward treatment-seeking, readiness for change, and technology readiness and acceptance (see Measures).

Next, participants were provided a brief description of chatbots (ie, what they are, common examples of chatbots, and their utility) and invited to either connect to a prototype chatbot (called “Tabatha”) designed to screen users for psychological distress and provide mental health resources and referrals, or end study participation; compensation was not linked to chatbot use. For participants declining to use the chatbot, the reason for their decision was solicited by the choices: “I have no interest in chatbots,” “I do not have the time to use a chatbot,” “I do not know what a chatbot is,” “I do not need mental health services,” “I prefer speaking to a human about my mental health.” An “other” option was also available so participants could provide additional reasons.

**Figure 2:**
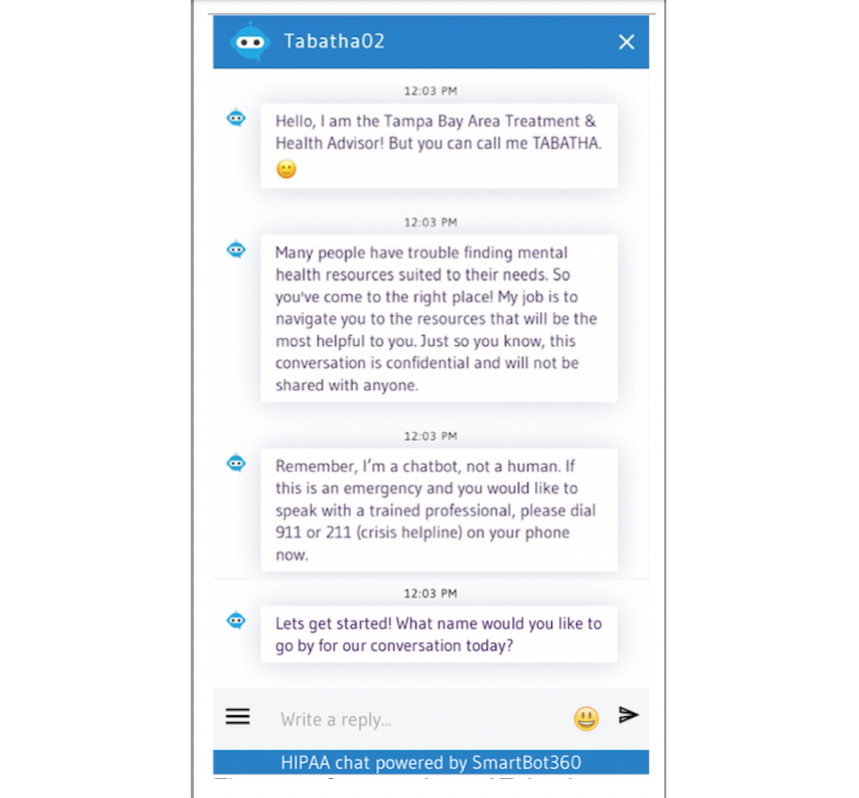
A screenshot of the Tabatha chatbot introduction received by each participant. HIPAA: Health Insurance Portability and Accountability Act.

**Figure 3:**
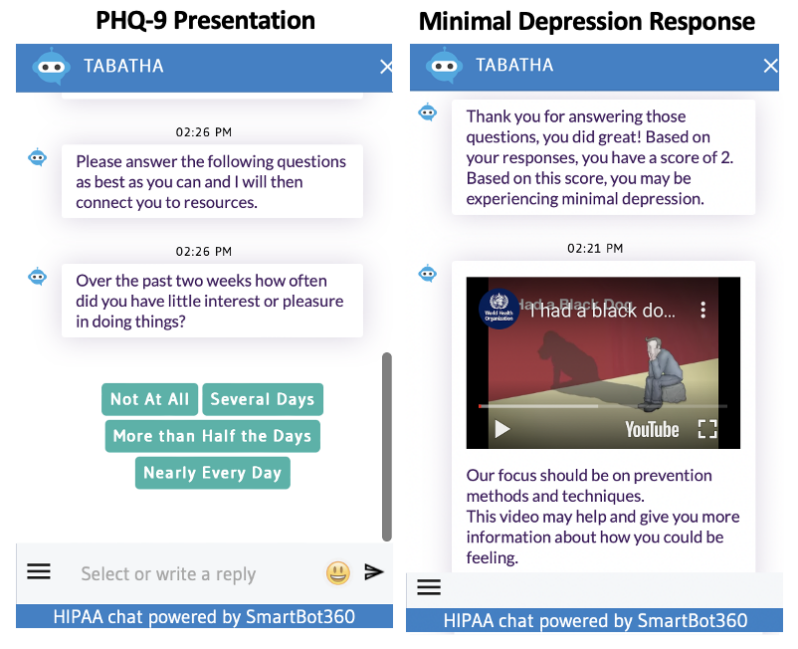
Screenshots of PHQ-9 chatbot presentation and minimal depression score resources presentation. HIPAA: Health Insurance Portability and Accountability Act; PHQ-9: Patient Health Questionnaire.

Participants agreeing to use the chatbot clicked on a link in this study’s questionnaire that opened a separate internet browser window in which the Tabatha chatbot appeared with text reading, “Hello, my name is Tabatha… What name would you like to go by for our conversation today?” (Figure 2). Users could provide any name by which they wished to be identified. Tabatha then greeted the participant using their name and stated that its purpose was to provide mental health screening and resource navigation, making clear that—despite the human-like nature of the text messages—it was a computer program and not a human responding. Further, Tabatha provided users with phone numbers for emergency services if they were experiencing a crisis. Next, Tabatha administered the Patient Health Questionnaire-9 (PHQ-9), a widely used, 9-question depression screener that scores the severity of depressive symptoms on a score of 0 (none) to 27 (severe) [23]. Participants were then provided with their PHQ-9 score and an explanation of its meaning (see screenshot of PHQ-9 screener and results presentation in Figure 3 [24-27]) and provided mental health resources commensurate to their level of distress based upon mental health professional feedback. Table 1 provides the interpretation of PHQ-9 scores in terms of the level of distress, the chatbot’s response based on the PHQ-9 scores, and resources provided based on distress levels.

Participants who chose to use the chatbot were asked to respond to four acceptability statements adapted from the Acceptability of Intervention Measure [28,29]. The acceptability statements included, “this mental health chatbot is appealing to me,” “this mental health chatbot meets my approval,” “I welcome mental health screening using a chatbot,” “I like this mental health chatbot,” and “I will use a chatbot like this in the future.” Finally, Tabatha asked participants if they would like to receive a single follow-up message 2 weeks later asking whether the referrals provided were used. For participants declining Tabatha’s follow-up message, study participation ended, while participants accepting the follow-up message were asked to provide a mobile telephone number capable of receiving SMS messages (this was necessary because, up until this point, the use of Tabatha was anonymous; a telephone number allowed for direct communication with the participant’s mobile phone). Further, 2 weeks later, Tabatha contacted participants who provided a mobile number, asking whether mental health resources were used.

**Table 1:** PHQ-9^a^ scores and corresponding responses and resources utilized in a cross-sectional survey examining the feasibility and acceptability of a mental health screening and referral chatbot among a sample of US adults.

PHQ-9 score range	Depression level	Tabatha response	Resources provided
0-4	Minimal	Thank you for answering those questions, you did great! Based on your responses, you have a score of ____. Based on this score, you may be experiencing none or minimal depression symptoms.	Our focus should be on prevention methods and techniques. This resource may help you recognize symptoms of depression should you experience them in the future [24]. This video may help and give you more information about how you could be feeling [25].
5-9	Mild	Thank you for answering those questions, you did great! Based on your responses, you have a score of ____. Based on this score, you may be experiencing mild depression symptoms.	I would like to offer you some resources and tips to help address how you have been feeling. To learn more about what you may be experiencing and to find some helpful tips for reducing depression symptoms, visit [24].
10-14	Moderate	Thank you for answering those questions, you did great! Based on your responses, you have a score of ____. Based on this score, you may be experiencing moderate depression symptoms.	I would like to offer you some resources and tips to help address how you have been feeling. To learn more about what you may be experiencing and to find some helpful tips for reducing depression symptoms, visit [24].
15-19	Moderately severe	Thank you for answering those questions, you did great! Based on your responses, you have a score of ____. Based on this score, you may be experiencing moderately severe depression symptoms.	The Crisis Text Line may be able to further assist you with the symptoms you are experiencing. Text HOME to 741741 and a team member will support you and connect you to the appropriate resources. Or if you would prefer to speak with a person on the phone, dial 1-800-662-HELP (4357).
20-27	Severe	Thank you for answering those questions, you did great! Based on your responses, you have a score of ____. Based on this score, you may be experiencing severe depression symptoms.	I would like to connect you to a Crisis Support Lifeline. Please follow this link for support [26]. You can also text HOME to 741741 if you would prefer. An additional resource for you can be found here [27].

^a^PHQ-9: Patient Health Questionnaire.

### Ethical Considerations

This study was reviewed and approved by the University of South Florida’s Institutional Review Board (IRB; STUDY002142). The IRB determined that this research was exempt from IRB oversight. Before completing the survey, participants were presented with an informed consent document. This study was granted a waiver of signed informed consent and participants were asked to acknowledge consent to participate in this study by clicking a radial box within the survey. All participants were compensated US $5.00 (the standard rate for MTurk users) for study participation regardless of chatbot use per Figure 1. The US $5.00 compensation was paid to the MTurk user through the MTurk platform. Study data were deidentified and stored in a password-protected [30] folder that could only be accessed by members of this study’s team.

### Measures

#### Symptoms

Symptoms were measured within the chatbot session using the PHQ-9, a 9-item depression symptom checklist [23]. The 9 items are scored from 0 to 3 on a Likert scale of “not at all” to “nearly every day.” The PHQ-9 internal reliability and test-retest reliability are high [23]. The PHQ-9 is meant to be self-administered, making the chatbot administration of this screening tool a reasonable adaptation. In the survey completed before initiating the chatbot session, symptoms were assessed using the *Diagnostic and Statistical Manual of Mental Disorders, Fifth Edition* (*DSM-5*) Level 1 Cross-Cutting Symptom Measure [31], a self-rated measure assessing mental health domains that are important across psychiatric diagnoses. This adult version of the measure contains 23 questions assessing 13 psychiatric domains, including depression, anger, mania, anxiety, somatic symptoms, suicidal ideation, psychosis, sleep problems, memory, repetitive thoughts and behaviors, dissociation, personality functioning, and substance use. Each item asks how much or how often the individual has been bothered by the specific symptom during the past 2 weeks. The measure was found to be clinically useful and to have good test-retest reliability in the *DSM-5* field trials that were conducted in adult clinical samples across the United States and Canada [31].

#### Readiness for Change

The URICA (University of Rhode Island Change Assessment) was used to measure the stage of change (precontemplation, contemplation, action, and maintenance) of a user of Tabatha. This 32-item scale uses a Likert scale from 1 (strongly disagree) to 5 (strongly agree) to answer statements describing how a person might feel toward treatment or approaching problems [32]. The URICA has good reliability with α from .79 to .89 and good construct validity supported through factor analyses [32].

#### Attitudes Toward Treatment-Seeking

Attitudes toward treatment-seeking were measured using an adapted version of the ATMHT (Attitudes Toward Mental Health Treatment) scale [33]. The scale was adapted from Fischer and Turner's Attitudes Toward Seeking Professional Psychological Help scale [34] to update language and culturally meaningful items. This 2-factor scale (beneficial attitudes and pessimistic attitudes toward mental health services) has demonstrated adequate internal consistency (beneficial attitudes toward mental health services α=.84; pessimistic attitudes toward mental health services α=.79), reliability, and validity. The ATMHT scale contains 20 items assessed on a 4-point Likert scale (4=strongly agree).

#### Perceived Stigma

We measured perceived stigma using the perceived devaluation-discrimination scale, a 12-item instrument asking participants to indicate the extent to which they agree with statements indicating that most people devalue individuals with mental illness (eg, most people feel that entering a psychiatric hospital is a sign of a personal failure) [35]. Participants respond using a 6-point Likert scale (6=strongly disagree). Higher scores on the perceived devaluation-discrimination scale represent greater perceived stigma. The scale has demonstrated good reliability, with α ranging from .86 to .88 [35] and validity [36].

#### Label Avoidance

The Self-Stigma of Seeking Help (SSOSH) scale measured label avoidance [37]. The SSOSH is a 10-item measure asking participants to answer on a 5-point Likert scale (5=strongly agree). The SSOSH has been found to have good internal consistency (α=.91), and test-retest reliability (*r*=0.72) [37].

#### Technology Readiness and Acceptance

The TRAM (Technology Readiness and Acceptance Model) is a questionnaire that incorporates both the Technology Readiness Index and the Technology Acceptance Model for a deeper understanding of the use of technology and an individual’s readiness and acceptance [38,39]. All items are measured on a 7-point Likert scale (7=strongly agree). The TRAM has good reliability for each of the subscales (optimism, α=.95; innovativeness, α=.95; discomfort, α=.90; insecurity, α=.92; perceived usefulness, α=.95; perceived ease of use and use intention, α=.92) [38]. The TRAM has been found to have adequate model fit.

#### Acceptability

Acceptability of the chatbot following use was measured using four items adapted from the Acceptability of Intervention Measure.

### Analytic Approach

Feasibility was modeled descriptively based on willingness to use the chatbot. We used *χ*^2^ tests to examine the relationship between categorical demographic variables and the dichotomous outcome variable, willingness to use chatbot (yes or no). Continuous variables were examined as predictors of willingness to use the chatbot via correlations and logistic regression. Acceptability data were examined using descriptive statistics.

## Results

### Overview

Guidelines for Transparent Reporting of Evaluations with Nonrandomized Designs were followed. This study enrolled 640 individuals; 329 individuals were included in the analyses. Correlations between all variables for the total sample can be found in Table S1 of Multimedia Appendix 1. Further, 80 participants were excluded from analyses as they answered 1 of the 3 attention check questions dispersed throughout the survey incorrectly. These questions were included to ensure that participants were paying attention to this study and not just selecting random responses and that the participant was not a bot. These 3 items consisted of a question asking the participant to spell the word “horse” backward, a question asking the participant to describe a picture of a picnic scene, and a question asking the participant to respond with “somewhat agree.” In total, 71 participants were excluded because they responded to the survey more than once, even given explicit instructions that they could only respond to the survey 1 time. Further, 160 individuals were excluded from analyses as they had taken less than 10 minutes to complete the survey. Table 2 presents the demographic characteristics of participants.

**Table 2:** Demographic characteristics of a sample of US adults from a cross-sectional survey examining the feasibility and acceptability of a mental health screening and referral chatbot^a^.

				Total		Agreed to chatbot use		Declined chatbot use		Inferential statistics								
										Chi-square (*df*)			*P* value			Cramér *V*		
**Sex, n (%)**											139.3 (2)			.19			0.10	
		Female		119 (36.2)		85 (71.4)		34 (28.6)										
		Male		209 (63.5)		137 (65.6)		72 (34.4)										
		Intersex		0 (0)		—^b^		—										
		None of these		0 (0)		—		—										
		Prefer not to say		1 (0.3)		—		—										
**Gender, n (%)**											5.98 (4)			.20			0.14	
		Man		206 (62.6)		135 (65.5)		71 (34.5)										
		Woman		117 (35.6)		84 (71.8)		33 (28.2)										
		Nonbinary		1 (0.3)		—		—										
		Transgender		1 (0.3)		—		—										
		None of these		2 (0.6)		—		—										
		Prefer not to say		2 (0.6)		—		—										
**Race, n (%)**											11.82 (5)			.04			0.19	
		Asian^c^		12 (3.6)		5 (41.7)		7 (58.3)										
		Black or African American^d^		45 (13.7)		34 (75.6)		11 (24.4)										
		White^d^		262 (79.6)		179 (68.3)		83 (31.7)										
		American Indian or Alaskan Native		1 (0.3)		—		—										
		Native Hawaiian or Pacific Islander		0 (0)		—		—										
		Other		1 (0.3)		—		—										
		Do not know		0 (0)		—		—										
		Prefer not to answer		2 (0.6)		—		—										
		Missing		6 (1.8)		—		—										
**Ethnicity, n (%)**											13.16 (2)			<.001			0.20	
		Hispanic or Latino^c^		81 (24.6)		67 (82.7)		14 (17.3)										
		Not Hispanic or Latino^d^		247 (75.1)		155 (62.8)		92 (37.2)										
		Do not know		0 (0)		—		—										
		Prefer not to answer		1 (0.3)		—		—										
**Marital status, n (%)**											11.40 (7)			.12			0.19	
		Married		222 (67.5)		160 (72.6)		62 (27.9)										
		Divorced		15 (4.6)		9 (60)		6 (40)										
		Never married		76 (23.1)		44 (58.7)		31 (41.3)										
		A member of an unmarried couple		11 (3.3)		6 (54.5)		5 (45.5)										
		Widowed		2 (0.6)		—		—										
		Separated		1 (0.3)		—		—										
		Prefer not to answer		2 (0.6)		—		—										
**Do you have dependents or children? n (%)**											10.94 (2)			<.01			0.18	
		Yes^c^		201 (61.1)		148 (73.6)		53 (26.4)										
		No^d^		124 (37.7)		73 (58.9)		51 (41.1)										
		Prefer not to answer		4 (1.2)		—		—										
**Level of education**											11.20 (9)			.26			0.19	
		High school graduate		20 (6.1)		13 (65)		7 (35)										
		Some college, no degree		28 (8.5)		16 (57.1)		12 (42.9)										
		Associate degree		25 (7.6)		17 (68)		8 (32)										
		Master’s degree		58 (17.6)		129 (70.1)		55 (29.9)										
		Bachelor’s degree		184 (55.9)		42 (72.4)		16 (27.6)										
		No high school diploma		4 (1.2)		—		—										
	GED^e^ or equivalent		4 (1.2)		—		—											
		Professional school degree		2 (0.6)		—		—										
		Doctoral degree		2 (0.6)		—		—										
		Do not know		0 (0)		—		—										
		Prefer not to answer		2 (0.6)		—		—										
**Employment**											9.82 (1)			.007			0.17	
		Employed		299 (90.9)		208 (69.6)		91 (30.4)										
		Unemployed		27 (8.2)		14 (51.9)		13 (48.1)										
		Prefer not to answer		3 (0.9)		—		—										
**Do you have health insurance coverage?**											12.49 (3)			<.01			0.20	
		Yes^c^		273 (83)		195 (71.4)		78 (28.6)										
		No^d^		49 (14.9)		24 (49)		25 (51)										
		Prefer not to answer		7 (2.1)		—		—										

^a^Cell counts <5 not presented for *χ*^2^ tests.

^b^not available.

^c^There was a statistically significant difference in the proportion of individuals agreeing to use the chatbot versus not agreeing between this category and those marked with “d.”

^d^There was a statistically significant difference in the proportion of individuals agreeing to use the chatbot versus not agreeing between this category and those marked with “c.”

^e^GED: General Educational Development.

### Feasibility

Table 2 provides the proportions of participants willing to use the chatbot by demographic characteristics. Of the 329 individuals included in the analyses, 222 (67.4%) agreed to use the chatbot. In terms of racial and ethnic variables, we found a significantly greater proportion of Black and White versus Asian participants (n=319, *χ*^2^_5_=11.82; *P*<.05) and a greater proportion of Hispanic versus non-Hispanic participants agreed to use the chatbot (n=328, *χ*^2^_2_=13.16; *P*<.001). We found that other demographic variables also contributed, with a significantly larger proportion of participants who had dependents versus no dependents (n=325, *χ*^2^_2_=10.94; *P*<.01), those with insurance versus without insurance (n=322, *χ*^2^_3_=12.49; *P*<.01), and those who were employed versus unemployed (n=326, *χ*^2^_2_=9.82; *P*<.01) agreeing to use the chatbot.

Table 3 presents the mental health characteristics of participants and the proportions of participants willing to use the chatbot by mental health characteristics. Current mental health variables were related to agreeing to use the chatbot with a significantly greater proportion of those who were currently receiving treatment versus not currently receiving treatment (n=325, *χ*^2^_2_=15.13; *P*=.001), those with a diagnosis versus no diagnosis (n=322, *χ*^2^_2_=9.89; *P*<.01), and those currently distressed versus not currently distressed (n=312, *χ*^2^_2_=21.69; *P*<.001) agreeing to use the chatbot. All effect sizes for these findings were small, with Cramér *V* ranging between 0.17 and 0.26. For individual effect sizes, refer to Table 3.

Correlations between continuous variables and willingness to use the chatbot for the entire sample and for those individuals who indicated that they were currently experiencing distress can be found in Multimedia Appendix 1. Table 4 displays the results of forward stepwise logistic regressions with willingness to use the chatbot as the outcome variable for the overall sample as well as for those individuals who indicated that they were currently experiencing distress. Significant correlates of chatbot use were included as predictor variables in the regression models. For the overall sample, the perceived usefulness of the chatbot and symptoms significantly predicted chatbot use, with greater perceived usefulness and greater symptoms predicting the likelihood that the participant agreed to use the chatbot. For the distressed sample, higher levels of label avoidance significantly predicted a greater likelihood of agreeing to use the chatbot.

**Table 3:** Mental health characteristics of a sample of US adults from a cross-sectional survey examining the feasibility and acceptability of a mental health screening and referral chatbot^a^.

			Total		Agreed to chatbot use		Declined chatbot use		Inferential statistics						
									Chi-square (*df*)			*P* value		Cramér *V*	
**Are you currently receiving mental health treatment (ie, therapy, medication, and peer support)?**										15.13 (2)		.001			0.21
	Yes^b^	109 (33.1)		89 (81.7)		20 (18.3)									
	No	216 (65.7)		131 (60.6)		85 (39.4)									
	Prefer not to answer	4 (1.2)		—^c^		—									
**Have you ever received a diagnosis of a mental health condition from a doctor or counselor?**										9.89 (2)		<.01			0.17
	Yes^b^	116 (35.3)		91 (78.4)		25 (21.6)									
	No^d^	206 (62.6)		127 (61.7)		79 (38.3)									
	Unsure	7 (2.1)		4 (57.1)		3 (42.9)									
	Prefer not to answer	0 (0)		—		—									
**Are you currently experiencing any mental distress?**										21.69 (2)		<.001			0.26
	Yes^b^	83 (25.3)		73 (88)		10 (12)									
	No^d^	229 (69.6)		140 (61.1)		89 (38.9)									
	Unsure	17 (5.2)		9 (52.9)		8 (47.1)									
	Prefer not to answer	0 (0)		—		—									
*DSM-5*^e^ Cross Cutting Symptoms, mean (SD)			30.0 (22.4)		—		—								

^a^Cell counts <5 not presented for *χ*^2^ tests.

^b^There was a statistically significant difference in the proportion of individuals agreeing to use the chatbot versus not agreeing between this category and those marked with “d.”

^c^not available.

^d^There was a statistically significant difference in the proportion of individuals agreeing to use the chatbot versus not agreeing between this category and those marked with “b.”

^e^*DSM-5: Diagnostic and Statistical Manual of Mental Disorders, Fifth Edition*.

**Table 4:** Predictors of chatbot use among a sample of US adults from a cross-sectional survey examining the feasibility and acceptability of a mental health screening and referral chatbot.

Variable			Estimate		SE (95% CI)		*P* value
**Full sample (N=329)^a^**							
	Intercept	–1.05		0.42		—^b^	
	Perceived usefulness	0.27		0.09 (1.10-1.56)		.002	
	*DSM-5* CC^c^ symptoms	0.02		0.01 (1.01-1.03)		.002	
**Distressed participants (n=83)^d^**							
	Intercept	–1.61		1.72		—	
	Label avoidance	0.13		0.06 (1.01-1.29)		.04	

^a^*χ*^2^_2_=28.10; *P*<.001.

^b^not available.

^c^*DSM-5 CC: Diagnostic and Statistical Manual of Mental Disorders, Fifth Edition cross cutting symptoms*.

^d^*χ*^2^_1_=4.16, *P*<.01.

### Acceptability

Of the 222 participants who agreed to use the chatbot, 168 (75.7%) participants completed the PHQ-9 screening questions in the chatbot, and 164 (73.9%) answered all of the acceptability items. The average depression score was 8.6 (SD 7.2), which constitutes mild depression among the sample of respondents who chose to complete the PHQ-9 screening within the chatbot session. Overall, participants agreed with the acceptability of the chatbot. The mean acceptability score was 19.8 (SD 4.2).

Participants who chose not to use the chatbot (n=107) indicated that they chose not to do so because they did not believe they needed mental health services (n=40), they had no interest in chatbots (n=36), they preferred to speak to a human about their mental health (n=33), they did not have time to use a chatbot (n=20), they did not know what a chatbot is (n=1), or they indicated some other reason (n=8). Frequencies of the reasons participants selected for not using the chatbot can be found in Table S2 of Multimedia Appendix 1. If they agreed to use the chatbot, participants were asked if they would be willing to provide their phone numbers within the chatbot session so that the chatbot could follow up with them in 2 weeks to see if any resources they received were used and whether they were helpful. Further, 63 (28.4% of those participants who agreed to use the chatbot) individuals agreed to provide their phone numbers. Furthermore, 56 (25.2% of those who agreed to use the chatbot) individuals agreed to allow the chatbot to follow up with them in 2 weeks; however, not all of these individuals provided a phone number for follow-up. The reasons participants provided for not wanting to provide their phone numbers can be found in Table S3 of Multimedia Appendix 1, along with frequencies for each response. Reasons provided for not wanting to provide one’s phone number included “my contact information is private” (n=61), “I do not give my number to strangers” (n=34), “I do not want unsolicited calls” (n=32), “it does not feel confidential” (n=20), “I do not want to give my number to a robot” (n=20), “I do not trust chatbots” (n=3), or “another reason not listed” (n=8).

Of the 56 individuals who received a follow-up text at 2 weeks, 9 (16.1%) individuals clicked on the link in the follow-up text to engage with the follow-up chatbot. Further, 4 (7.1%) individuals stated that they did not use the resources. Reasons included “I didn’t have time to look them over” (N=2) and “I didn’t feel like I needed them” (N=1). Of the 5 people who reported using the resources, all stated they were helpful. Further, 1 participant reported that the resources they received “…really help[ed] to move on to the next stage.” Another stated, “it was good advice.”

## Discussion

### Principal Findings

We found in this exploratory study that a chatbot designed to screen for mental distress and refer to appropriate resources matching one’s distress level was feasible and acceptable among a convenience sample of US adults. Moreover, label avoidance was the single significant, positive predictor of chatbot use among distressed participants. Most participants (n=222, 67.4%) were willing to try the chatbot, indicating that using a chatbot to screen for mental distress and connect patients or participants to resources is feasible. Of the 222 individuals who agreed to use the chatbot, 168 (75.7%) completed the PHQ-9 screening within the chatbot session, and 164 (73.9%) persisted in completing the acceptability questionnaire at the end of the chatbot interaction. Again, these findings speak to the feasibility of using chatbot technology to facilitate mental health screening and referral.

A small number of participants agreed to provide their phone number within the chatbot session for follow-up purposes and a small subgroup of these participants clicked on the link within the follow-up text they received 1 month after completing the initial survey and chatbot session. Understanding the reasons for not using the chatbot and not providing one’s phone number are important to modify the chatbot and develop messaging and marketing around the chatbot that will address potential user concerns. The most cited reasons for not using the chatbot included a perception that one did not need mental health services, did not have an interest in chatbots, and preferred to speak with a human about one’s mental health. To address some of these points, future iterations of the chatbot might include attention to the marketing of the chatbot, incorporating messaging around the need for mental health screening for preventative care as well as helping potential users to understand that the chatbot is not meant to replace human interaction and screening may result in referral to a human such as a peer or a licensed mental health professional. The top reasons for not providing one’s phone number to enable follow-up included a desire to keep one’s contact information private, a policy that one does not share one’s number with strangers, and not wanting solicited calls. Future iterations of this chatbot may entertain other possible mechanisms for follow-up as an alternative to providing a phone number and more clear messaging about how a phone number would be used (ie, only for a follow-up text as opposed to a call).

Overall, the participants who did agree to try the chatbot found it acceptable. The feasibility and acceptability findings of this study are in line with recent work by Shah and colleagues [40] to develop and iteratively test a chatbot to screen users for eating disorders and refer them to care. Demographic predictors of chatbot use included being White or Black or African American, identifying as Hispanic or Latino, having dependents, having insurance coverage, being employed, having used mental health services in the past, having received a diagnosis of a mental health condition, and reporting current distress. Taking these demographic characteristics into consideration when designing future iterations of the chatbot will be crucial to making sure the chatbot is available to those who need it, is culturally relevant and responsive, and is marketed appropriately. Positive correlates of chatbot use among the full sample included technology discomfort and insecurity, symptoms, beneficial and pessimistic attitudes toward treatment-seeking, perceived usefulness of the chatbot, and reported intentions to use the chatbot. Among the full sample, the only significant predictors of chatbot use were perceived stigma and *DSM-5* cross-cutting symptoms. Among those participants who endorsed current psychological distress, the only significant predictor of chatbot use was label avoidance.

### Limitations

This feasibility and acceptability study had limitations. First, we recruited participants through Amazon’s MTurk, which limits the generalizability of these findings. Second, we did lose a substantial portion of our sample due to either inattention to or an unreasonably short time taken to complete the survey. On the one hand, this may lead one to question the integrity of the data provided by participants of MTurk. On the other hand, we have greater confidence in the quality of the data that we did include in our analyses due to the data-cleaning procedures that we applied. Finally, the effect sizes for our statistical tests (*χ*^2^ tests in particular) are small. A replication of this study with a larger sample is called for to confirm these findings.

### Conclusions

This study found that label avoidance was the single significant, positive predictor of chatbot use among distressed participants. The existing research literature suggests that label avoidance, the desire to avoid mental health services to avoid the stigmatized label of mental illness, is a significant negative predictor of care-seeking [41,42]. Therefore, our finding regarding label avoidance and chatbot use has significant public health implications in terms of facilitating access to mental health resources and stigma reduction programs and messaging. Those who are high on label avoidance are not likely to seek care in a community mental health clinic, yet they are likely willing to engage with a mental health chatbot, participate in mental health screening, and receive mental health resources within the chatbot session.

In 2020, among the 52.9 million adults living in the United States with any mental illness, less than half (46.2%) received mental health services in the past year [43]. This technology-facilitated approach to connecting people with mental health resources holds promise to reach the nearly 54% of US adults living with a mental illness who are currently going without care and presents an opportunity for intervention as well. Research suggests that many adults in the United States living with the most common mental health condition, major depressive disorder, are receiving treatment from their primary care provider [44]. This is an important consideration for future work with this chatbot for a couple of reasons. First, primary care offices may be a deployment site for a chatbot like the one we tested. This could help ensure behavioral health screening and referral and minimize demand on primary care providers. Second, the use of technology for screening and referral in primary care settings may help to eliminate the possible effects of clinician bias on behavioral health screening and referral [45].

Stigma reduction interventions (ie, videos of people sharing their stories of mental health challenges, service usage, and recovery) might be delivered via the chatbot to support those who need it to seek mental health services from a licensed clinician. Research suggests that video-based contact is an effective means of reducing the stigma surrounding mental illness [46]. Additionally, motivational interviewing strategies might be used within the chatbot session to move users through the transtheoretical model of health behavior change [47], toward a decision to seek evidence-based treatments. Others have demonstrated the possibility of deploying motivational interviewing conversational sequencing using chatbot technology for stress management [48] and smoking cessation [49]. Chatbot technology may prove to be a way to engage those in care who have previously avoided treatment due to stigma, which could have a significant public health impact by way of shrinking the mental health treatment gap.

The future vision for Tabatha, given the feasibility and acceptability of the prototype chatbot, is as a conversational agent aimed at assuming the service delivery tasks of screening, referral, and stigma reduction. Tabatha might be deployed in various settings with diverse populations, and refer to a plethora of existing resources, including other conversational agents designed to deliver digital therapy (ie, reSET-O, Woebot, and Wysa). Future research should focus on examining Tabatha’s effectiveness in reducing stigma and navigating users to existing behavioral health resources of various intensities. The current Tabatha prototype is a rule-based chatbot, as opposed to a machine-learning chatbot [50]. Future research might consider how machine learning might be used to facilitate screening, referral, and stigma reduction through Tabatha.

## Appendix

Multimedia Appendix 1 Tables with additional statistics.

## Abbreviations

ATMHT: Attitudes Toward Mental Health Treatment
*DSM-5*: *Diagnostic and Statistical Manual of Mental Disorders, Fifth Edition*
IRB: Institutional Review Board
MTurk: Amazon Mechanical Turk
PHQ-9: Patient Health Questionnaire-9
SBIRT: Screening, Brief Intervention, and Referral
SSOSH: Self-Stigma of Seeking Help
TRAM: Technology Readiness and Acceptance Model
URICA: University of Rhode Island Change Assessment

## Data Availability

The data sets generated and analyzed during this study are available from the corresponding author upon reasonable request.
