# Supplementary material for: Mental Distress, Label Avoidance, and Use of a Mental Health Chatbot: Results From a US Survey
Source: JMIR Form Res. 2024 Apr 12;8:e45959. doi: 10.2196/45959 (PMC11053397; doi:10.2196/45959)
Supplement: Multimedia Appendix 1 [file formative_v8i1e45959_app1.docx]

**Table S1.** Correlations for full sample (N=329) and distressed participants (N=83) from a cross-sectional survey examining the feasibility and acceptability of a mental health screening and referral chatbot among US adults.

|  | Variables | 1 | 2 | 3 | 4 | 5 | 6 | 7 | 8 | 9 | 10 | 11 | 12 | | 13 | 14 |
| --- | --- | --- | --- | --- | --- | --- | --- | --- | --- | --- | --- | --- | --- | --- | --- | --- |
| **1.** | ***Willingness to Engage*** |  |  |  |  |  |  |  |  |  |  |  |  |  | |  |
|  | Full Sample | - |  |  |  |  |  |  |  |  |  |  |  |  | |  |
|  | Distressed | - |  |  |  |  |  |  |  |  |  |  |  |  | |  |
| **2.** | ***Readiness for Change*** |  |  |  |  |  |  |  |  |  |  |  |  |  | |  |
|  | Full Sample | N/A | - |  |  |  |  |  |  |  |  |  |  |  | |  |
|  | Distressed | 0.1 | - |  |  |  |  |  |  |  |  |  |  |  | |  |
| **3.** | ***Optimism*** |  |  |  |  |  |  |  |  |  |  |  |  |  | |  |
|  | Full Sample | 0.1 | N/A | - |  |  |  |  |  |  |  |  |  |  | |  |
|  | Distressed | -0.1 | 0.6** | - |  |  |  |  |  |  |  |  |  |  | |  |
| **4.** | ***Innovation*** |  |  |  |  |  |  |  |  |  |  |  |  |  | |  |
|  | Full Sample | 0 | N/A | 0.5** |  |  |  |  |  |  |  |  |  |  | |  |
|  | Distressed | 0.1 | 0.6** | 0.7** | - |  |  |  |  |  |  |  |  |  | |  |
| **5.** | ***Discomfort*** |  |  |  |  |  |  |  |  |  |  |  |  |  | |  |
|  | Full Sample | .01* | N/A | 0 | 0.03** | - |  |  |  |  |  |  |  |  | |  |
|  | Distressed | 0.1 | 0.8** | 0.3** | 0.5** | - |  |  |  |  |  |  |  |  | |  |
| **6.** | ***Insecurity*** |  |  |  |  |  |  |  |  |  |  |  |  |  | |  |
|  | Full Sample | .02** | N/A | -0.2 | 0 | 0.5** | - |  |  |  |  |  |  |  | |  |
|  | Distressed | 0.2 | 0.6** | 0.4** | 0.5** | 0.6** | - |  |  |  |  |  |  |  | |  |
| **7.** | ***DSM CC Symptoms*** |  |  |  |  |  |  |  |  |  |  |  |  |  | |  |
|  | Full Sample | .02** | N/A | -0.1 | 0.2** | 0.6** | 0.5** | - |  |  |  |  |  |  | |  |
|  | Distressed | 0.2 | 0.7** | 0.4** | 0.4** | 0.6** | 0.5** | - |  |  |  |  |  |  | |  |
| **8.** | ***Perceived Stigma*** |  |  |  |  |  |  |  |  |  |  |  |  |  | |  |
|  | Full Sample | -.01* | N/A | 0 | -0.1 | -0.1* | .01 | -0.1* | - |  |  |  |  |  | |  |
|  | Distressed | -0.1 | -0.2 | -0.1 | -0.4** | -0.2 | -0.2 | -0.2* | - |  |  |  |  |  | |  |
| **9.** | ***Label Avoidance*** |  |  |  |  |  |  |  |  |  |  |  |  |  | |  |
|  | Full Sample | .01 | N/A | -0.2** | -0.1 | 0.3** | 0.4** | 0.4** | 0.2** | - |  |  |  |  | |  |
|  | Distressed | 0.2* | 0.2 | -0.1 | -0.1 | 0.4** | 0.3** | 0.4** | -0.1 | - |  |  |  |  | |  |
| **10.** | ***BA*** |  |  |  |  |  |  |  |  |  |  |  |  |  | |  |
|  | Full Sample | 0.2* | N/A | 0.4** | 0.4** | 0.3* | 0.1* | 0.1** | -0.2** | -0.4** | - |  |  |  | |  |
|  | Distressed | 0 | 0.6** | 0.5 | 0.7** | 0.4** | 0.4** | 0.4** | -0.2 | -0.1 | - |  |  |  | |  |
| **11.** | ***PA*** |  |  |  |  |  |  |  |  |  |  |  |  |  | |  |
|  | Full Sample | 0.2* | N/A | -0.1 | 0.1** | 0.6* | 0.6* | 0.7** | 0 | 0.6** | 0 |  |  |  | |  |
|  | Distressed | 0.2 | 0.7** | 0.2** | 0.3** | 0.6** | 0.7** | 0.7** | -0.2 | 0.4** | 0.4** | - |  |  | |  |
| **12.** | ***Perceived Ease of Use*** |  |  |  |  |  |  |  |  |  |  |  |  |  | |  |
|  | Full Sample | 0.1 | N/A | 0.5** | 0.4** | -0.1 | -0.1 | -0.2** | 0 | -0.2** | 0.3** | -0.1 | - |  | |  |
|  | Distressed | 0 | 0.2 | 0.5** | 0.5** | 0.1 | 0.2** | 0.3* | -0.2* | -0.1 | 0.4** | 0.1 | - |  | |  |
| **13.** | ***Perceived Usefulness*** |  |  |  |  |  |  |  |  |  |  |  |  |  | |  |
|  | Full Sample | 0.2** | N/A | 0.3** | 0.4** | 0.4** | 0.2** | 0.3** | -0.2** | 0 | 0.4** | 0.3** | .03** | - | |  |
|  | Distressed | 0.2 | 0.7** | 0.5** | 0.6** | 0.5** | 0.5** | 0.4** | -0.4** | 0 | 0.5** | 0.4** | 0.3** | - | |  |
| **14.** | ***Intent to Use*** |  |  |  |  |  |  |  |  |  |  |  |  |  | |  |
|  | Full Sample | 0.2** | N/A | 0.2** | 0.4** | 0.6** | 0.3** | 0.5** | -0.2** | 0.1* | 0.3** | 0.4** | 0.1** | 0.7** | | - |
|  | Distressed | 0.2* | 0.7** | 0.3** | 0.5** | 0.6** | 0.5** | 0.6** | -0.3* | 0.2* | 0.5** | 0.5** | 0.3** | 0.6** | | - |

* p<0.5

** p<0.1

Note: Readiness for Change was only measured for currently distressed participants.

**Table S2.** Reasons provided for not using the chatbot from a cross-sectional survey examining the feasibility and acceptability of a mental health screening and referral chatbot among US adults.

| **Reason** | **N (%)*** |
| --- | --- |
| I do not need mental health services. | 40 (37.4) |
| I have no interest in chatbots. | 36 (33.6) |
| I prefer speaking with a human about my mental health. | 33 (30.8) |
| I do not have time to use a chatbot. | 20 (18.7) |
| Other | 8 (7.5) |
| I do not know what a chatbot is. | 1 (0.9) |

*107 participants declined the opportunity to use the chatbot.

Note: Participants were allowed to indicate more than one reason for choosing not to use the chatbot.

**Table S3.** Reasons for not providing phone number within chatbot from a cross-sectional survey examining the feasibility and acceptability of a mental health screening and referral chatbot among US adults.

| **Reason** | **N (%)*** |
| --- | --- |
| My contact information is private. | 61 (38.4) |
| I do not give my number to strangers. | 34 (21.4) |
| I do not want solicited calls. | 32 (20.1) |
| It does not feel confidential. | 20 (12.6) |
| I do not want to give my number to a robot. | 20 (12.6) |
| Another reason not listed. | 8 (5.0) |
| I do not trust chatbots. | 3 (1.9) |

*159 participants declined to provide their phone number.

Note: Participants were allowed to indicate more than one reason for choosing not to use the chatbot.
